# Effect of zinc hydroxychloride supplementation combined with an anticoccidial drug on *Eimeria tenella* infection in broiler chickens

**DOI:** 10.14202/vetworld.2023.675-680

**Published:** 2023-04-04

**Authors:** Tippayaporn Nonkookhetkhong, Thanyakorn Chalalai

**Affiliations:** Stress and Oxidation Stress in Animals Research Unit, Faculty of Veterinary Sciences, Mahasarakham University, Maha Sarakham, Thailand

**Keywords:** anticoccidial drug, broilers, *Eimeria tenella*, zinc hydroxychloride

## Abstract

**Background and Aim:**

*Eimeria tenella* is a causative agent of avian cecal coccidiosis resulting in bleeding, diarrhea, weight loss, high morbidity, and mortality in chickens. Zinc supplementation increases body weight gain, reduces mortality, and improves some immune response parameters of broilers infected with *E. tenella*. This study aimed to investigate the effects of zinc hydroxychloride (ZnOHCl) supplementation and ZnOHCl combined with an anticoccidial drug on *E. tenella* infection in broiler chickens.

**Materials and Methods:**

Forty one-day-old broilers were randomly divided into five groups, the study was replicated twice and had four chickens per replicate. Group 1 was an uninfected and unmedicated control group, and Group 2 was an infected but unmedicated control group. Group 3 was infected and treated with 120 mg/kg ZnOHCl, Group 4 was infected and medicated with 7 mg/kg toltrazuril (TOL), and Group 5 was infected and treated with 120 mg/kg ZnOHCl and 7 mg/kg TOL. Body weight gain, feed intake, and feed conversion ratio were monitored on days 15, 21, and 28. Oocyst shedding, hematological data, and lesion scores were analyzed on 7 days post-infection.

**Results:**

The average weight gain, feed intake, and packed cell volume of chickens treated with ZnOHCl and TOL were significantly higher than those of the infected and unmedicated controls (p ≤ 0.05). Lesion scores, oocyst output, and lymphocytes of the chickens treated with ZnOHCl and TOL were significantly lower than those of the infected and unmedicated controls (p ≤ 0.05).

**Conclusion:**

This study demonstrated that zinc supplementation alone reduced only oocyst output. However, growth performance, lesion scores, and oocyst output were affected by the combination of ZnOHCl and TOL supplementation. This suggests that ZnOHCl supplementation combined with an anticoccidial drug can improve growth performance and lessen the severity of *E. tenella* infection.

## Introduction

Coccidiosis is an important disease causing economic losses from poor body weight gain and high mortality in broilers [1]. It causes intestinal tissue damage, bloody diarrhea, and emaciation. The protozoan *Eimeria tenella* causes cecal or bloody coccidiosis. It multiplies in the cecum, which becomes distended with clotted blood, and in severe cases, it may also invade the intestine above and below the cecal junction [2, 3]. Coccidiosis in poultry is controlled using anticoccidial drugs such as sulfaquinoxaline, clopidol, nicarbazin, narasin, monensin, salinomycin, amprolium + ethopabate, and diclazuril. The most serious limitation to the drugs’ effectiveness is the development of resistance [4–6]. Therefore, many studies have used alternative methods, such as plant extracts, vitamins, and minerals, to control and treat the disease [7–9].

Zinc is an essential trace mineral with multiple roles in animal metabolism, including structural components, signaling mediators, and catalytic factors. It is a component of enzyme systems such as glutamic dehydrogenase, alcohol dehydrogenase, alkaline phosphatase, and RNA polymerase, which control DNA synthesis, normal growth, reproduction, and wound healing [10]. Zinc supplementation tends to increase body weight gain, reduce mortality, and enhance zinc deposition in the liver [11]. Moreover, it has been shown to reduce oxidative stress and improve some immune response parameters of broilers infected with *E. tenella* [12, 13]. Organic trace minerals such as chelated zinc and hydroxy trace minerals have been used. Zinc hydroxychloride (ZnOHCl) is a trace mineral that is stable in the digestive tract under low pH conditions and is effectively absorbed into the intestine [10, 14, 15].

This study aimed to investigate the effects of ZnOHCl supplementation and ZnOHCl combined with an anticoccidial drug on *E. tenella* infection in broiler chickens.

## Materials and Methods

### Ethical approval

The experiment was reviewed and approved by the Institutional Animal Care and Use Committee, Mahasarakham University (approval number: 033/2019).

### Study period and location

The study was conducted from January 2020 to November 2020 at Faculty of Veterinary Sciences, Mahasarakham University, Thailand.

### Parasites

*Eimeria tenella* oocysts were collected from the cecal contents of infected broiler chickens on farms in Roi Et province, Thailand, and maintained at the Faculty of Veterinary Sciences, Mahasarakham University, Thailand. The oocysts underwent sporulation in 2.5% (w/v) potassium dichromate at 30°C for 48 h. The sporulated oocysts were propagated in five commercial broiler chickens, 21 days old, by inoculation with 2 × 10^4^ sporulated oocysts [3]. Subsequently, the cecal contents were collected on 7 days post-infection (DPI) for experimental use.

### Effect of ZnOHCl and disinfectants on oocysts sporulation

Unsporulated oocysts (2 × 10^5^) were added to a solution of 20 mL of disinfectant and anticoccidial drugs containing 2% sodium hypochlorite (NaClO) (Kao Industrial, Chonburi, Thailand), 1% w/v iodine (Medic Phamaceutical company, Samut Sakhon, Thailand), 10% formalin (Qchemical, Bangkok, Thailand), 70% alcohol (Siribuncha Co., Ltd., Nonthaburi, Thailand), 0.25% potassium peroxymonosulfate (Antec International, Suffolk, UK), 2.5% toltrazuril (TOL) (Better Pharma, Lopburi, Thailand) from TOL 50 mg/mL, and 120 mg/mL ZnOHCl (XJ Biotech, Hunan, China). A tube without zinc and TOL served as a control. All products were diluted with distilled water. Incubation occurred at 25–30ºC for 24 h with three replicates per treatment. The number of sporulated oocysts was recorded and counted using a McMaster chamber through light microscopy.

### *In vivo* ZnOHCl and anticoccidial drug testing in chickens

#### Animals and experimental design

Forty, one-day-old commercial broiler chickens were obtained from a commercial hatchery. The chickens were housed in metal cages, under an open house system, and fed *ad libitum* during the experiment. At 15 days of age, the chickens were randomly divided into five groups, the study was replicated twice and had four chickens per replicate. The chickens in Group 1 were uninfected and unmedicated controls (UUC), and Group 2 was an infected but unmedicated control (IUC). Group 3 was infected and treated with 120 mg/kg of ZnOHCl, Group 4 was infected and medicated with 7 mg/kg of TOL, and Group 5 was infected and treated with 120 mg/kg of ZnOHCl plus 7 mg/kg of TOL (ZnOHCl + TOL). The chickens in Groups 3, 4, and 5 were treated from 15 days of age until 28 days and inoculated with 2 × 10^4^ sporulated oocysts at 21 days of age. On 28 days, 1 mL blood sample was collected from chickens in each group and the birds were then euthanized by cervical dislocation at 7 DPI. Cecal samples were collected from the euthanized chickens. During the experiment, chickens were fed *ad libitum*. Body weight gain and feed intake in each group were monitored on days 15, 21, and 28.

#### Lesion scoring

*Eimeria tenella* lesion scores were obtained by examining the intestinal lesions of euthanized chickens at 7 DPI. Scoring of the lesions was recorded as described previously by Johnson and Reid [16]: 0 = no gross lesions; 1 = mild lesions, very few scattered petechiae on the cecal wall; 2 = moderate lesions, more numerous lesions with blood in the cecal contents; 3 = severe lesions, large amounts of blood or cecal cores, cecal wall thickening; and 4 = extremely severe lesions, cecal wall was grossly distended with blood or large caseous cores.

#### Oocyst counting

The pooled fecal samples of each group were collected at 7 DPI. Oocysts were processed in a saturated sodium chloride solution and counted (oocysts/gram of feces) using a McMaster chamber under a light microscope.

#### Hematological analysis

Blood samples were collected from the wing vein and transferred immediately into a sterile tube containing an anticoagulant (ethylenediaminetetraacetic acid). Packed cell volume was measured using microhematocrit tubes and a hematocrit centrifuge, and a total white blood count was performed in a 1:200 dilution of blood in Natt and Herrick’s solution. Differential white blood cell counts were performed by preparing thin blood smears stained with Wright’s stain for the identification of lymphocytes, heterophils, eosinophils, monocytes, and basophils [17].

### Statistical analysis

Body weight gain, feed intake, feed conversion ratio (FCR), oocysts per gram of feces, and hematological data were analyzed using a one-way analysis of variance, and lesion scores were analyzed using the Kruskal–Wallis test with statistical package for the social sciences (SPSS) for Windows (SPSS Inc., Chicago). Statistical difference was considered significant at p ≤ 0.05.

## Results

### Effect of zinc and disinfectants on oocyst sporulation

**Figure-1:**
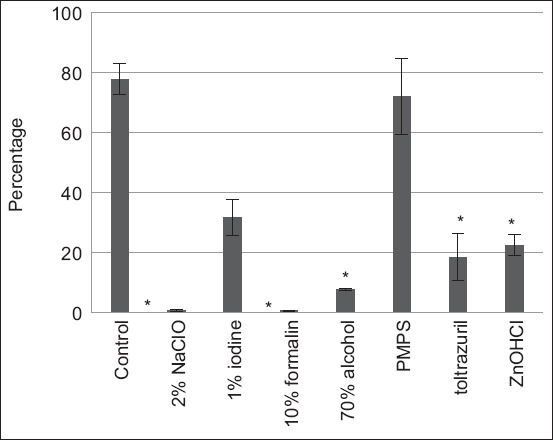
The mean (± standard deviation) of sporulated oocysts of experimental chickens.

Solutions containing 2% NaClO, 10% formalin, 70% alcohol, 2.5% TOL (50 mg/mL), and 58% ZnOHCl exhibited significant inhibitory effects on the sporulation of *E. tenella* oocysts compared to the negative control group. Marked inhibitory effects on sporulation were shown with solutions containing 2% NaClO, 10% formalin, and 70% alcohol. Solutions containing 1% iodine and 0.25% potassium peroxymonosulfate (Table-1 and Figure-1) had minimal inhibitory effects on sporulation.

**Table-1:** Effect of zinc and disinfectants on oocysts sporulation.

Item	Negative control	NaClO	1% Iodine	10% Formalin	70% Alcohol	Potassium peroxymonosulfate	Toltrazuril	Zinc hydroxychloride
Sporulated oocyst	155,733.0±44.99^a^	1,244.33±82.31^b^	63,284±13.11^a^	1,022.00±44.03^b^	15,269.00±71.62^b^	144,140.00±27.92^a^	36,644.00±57.78^b^	44,889.66±73.78^b^
% Sporulated oocyst	77.87±5.2^a^	0.63±0.3^b^	31.64±5.9^a^	0.51±0.2^b^	7.63±0.2^b^	72.07±12.6^a^	18.32±7.8^b^	22.44±3.6^b^
% Inhibition of sporulation	22.13±5.2^a^	99.37±0.3^b^	68.36±5.9^a^	99.49±0.2^b^	92.37±0.2^b^	22.93±12.6^a^	81.68±7.8^b^	77.56±3.6^b^

Different superscripts within a group differ significantly (p ≤ 0.05). NaClO=Sodium hypochlorite

### Weight gain, feed intake, and FCR

During 15–28 days of life when the experiment took place, the average weight gain of chickens treated with ZnOHCl + TOL was significantly higher than IUC (p ≤ 0.05). The average feed intake of chickens treated with TOL was significantly higher than IUC (p ≤ 0.05), and the average FCR of chickens treated with ZnOHCl + TOL was significantly lower than IUC (p ≤ 0.05) (Table-2).

**Table-2:** Weight gain, feed intake, and FCR of experimental chickens.

Group	Feed intake	Weight gain	FCR
UU	936.12±8.65^a^	305.00±7.07^a^	3.07±0.99^a^
IU	866.77±9.58^b^	185.00±7.07^b^	4.68±0.23^b^
ZnOHCl	866.50±2.12^b^	205.00±7.07^b^	4.23±0.13^b^
TOL	815.21±7.37^ab^	185.00±7.07^b^	4.41±0.20^b^
ZnOHCl+TOL	888.25±11.66^b^	233.75±8.83^ab^	3.80±0.19^a^

Different Superscripts Within A Group Differ Significantly (p ≤ 0.05). FCR=Feed Conversion Ratio, ZnOHCL=Zinc Hydroxychloride, TOL=Toltrazuril

### Lesion scores

The UUC group showed no clinical signs or lesions of the disease. After being inoculated with 2 × 10^4^ sporulated oocysts of *E. tenella*, all chickens in the infected groups had bloody diarrhea, but there was no mortality. The mean lesion scores of treated chickens in all groups were lower than the positive control, but the mean lesion scores of chickens treated with ZnOHCl + TOL were significantly lower than IUC (p ≤ 0.05) (Table-3).

**Table-3:** Lesion scores and oocyst output per gram of feces of experimental chickens.

Group	Lesion scores	Oocyst output (oocyst/g.)
UUC	0.00±0.00^a^	0.00^a^
IUC	3.50±0.57^b^	50687.22^ab^
ZnOHCl	3.00±0.81^b^	48582.33^b^
TOL	2.25±0.95^b^	34210.98^b^
ZnOHCl+TOL	2.00±1.45^a^	16266.76^b^

Different superscripts within a group differ significantly (p < 0.05). ZnOHCL=Zinc Hydroxychloride, TOL=Toltrazuril

### Oocyst output per gram of feces

The oocyst output of treated chickens in all groups was significantly lower than IUC (p ≤ 0.05) (Table-3).

### Hematologic values

**Figure-2:**
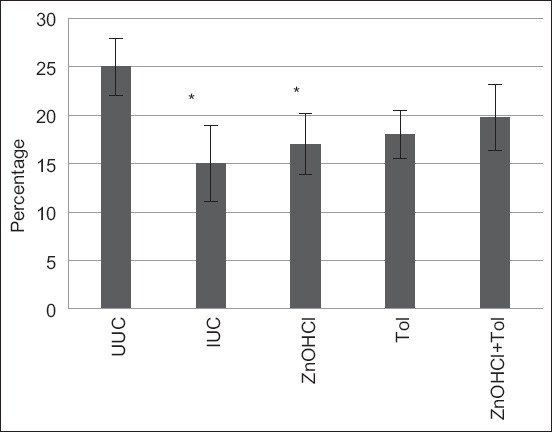
The mean (± standard deviation) of packed cell volume of experimental chickens (%).

**Figure-3:**
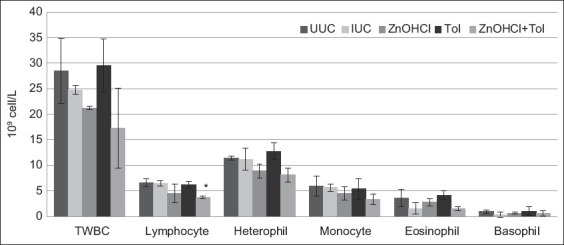
The mean (± standard deviation) of total white blood cell counts (TWBC) and differential white blood cell counts of experimental chickens (10^9^ cell/L).

The packed cell volume of chickens treated with ZnOHCl + TOL and TOL was significantly higher than that of IUC (p ≤ 0.05) (Table-4). The total number of leukocytes, heterophils, monocytes, eosinophils, and basophils of chickens in all groups was not significantly different from the IUC. The number of lymphocytes in chickens treated with ZnOHCl + TOL was significantly lower than IUC (p ≤ 0.05). All hematological values of chickens treated with ZnOHCl + TOL were lower than those of any other group (Table-4, Figures-2 and 3).

**Table-4:** Packed cell volume, TWBC, and different leucocyte counts (10^9^ cell/L) of experimental chickens.

Group	PCV	TWBC	Lymphocyte	Heterophil	Monocyte	Eosinophil	Basophil
UUC	25.00±2.94^a^	28.50±6.35^a^	6.56±0.75^a^	11.40±0.40^a^	5.91±1.99^a^	3.56±1.65^a^	0.92±0.35^a^
IUC	15.00±3.91^b^	24.75±0.86^a^	6.46±0.57^a^	11.20±2.14^a^	5.63±0.68^a^	1.53±1.09^a^	0.34±0.48^a^
ZnOHCl	17.00±3.16^b^	21.25±0.28^a^	4.47±1.87^a^	8.87±1.35^a^	4.51±1.29^a^	2.76±0.71^a^	0.63±0.17^a^
TOL	18.00±2.44^a^	29.5±5.19^a^	6.19±0.63^a^	12.75±1.58^a^	5.38±2.02^a^	4.13±0.83^a^	1.03±0.88^a^
ZnOHCl+TOL	19.75±3.40^a^	17.25±7.79^a^	3.66±0.25^b^	8.11±1.39^a^	3.36±1.03^a^	1.47±0.43^a^	0.60±0.56^a^

Different superscripts within a group differ significantly (p < 0.05). PCV=Packed cell volume, TWBC=Total white blood cell counts, UUC=Uninfected and unmedicated controls, IUC=Infected but unmedicated control, ZnOHCl=Zinc hydroxychloride, TOL=Toltrazuril

## Discussion

### Effect of disinfectants on oocyst sporulation

Solutions of 2% NaClO, 10% formalin, and 70% alcohol were able to inhibit more than 90% of sporulated oocysts. These results were similar to previous reports that ethanol, formalin, and NaClO showed effective inhibition of oocyst sporulation [18]. The anticoccidial effect of ZnOHCl was similar to that of TOL. The present findings indicate that ZnOHCl can suppress *E. tenella* oocyst sporulation.

### *In vivo* anticoccidial efficacy test

Zinc hydroxychloride supplementation was effective only on oocyst output reduction, and this finding was in agreement with data in a previous report [19]. However, growth performance, lesion scores, and oocyst output were affected by ZnOHCl supplementation combined with TOL. For healthy broilers, ZnOHCl improved production performance by increasing body weight gain, reducing mortality, and enhancing zinc deposition in the liver [11]. The current hematology results noted reductions in the erythrocyte count and hematocrit because blood loss from blood vessels was disrupted when the schizonts matured and the merozoites were released [2]. This was likely due to the packed cell volume of chickens treated with ZnOHCl + TOL, being significantly higher than IUC (p ≤ 0.05). Amadu in 2013 noted that *E. tenella* and *Eimeria brunetti* induced an increase in lymphocytes, monocytes, eosinophils, and heterophils [20], and leukocytosis is often associated with inflammatory diseases, infectious diseases, or stress [21]. Thus, the total white blood cell count of broilers receiving ZnOHCl + TOL being lower than the other groups might be due to less intestinal damage, as reflected in the lowest mean lesion score. This suggests that ZnOHCl + TOL supplementation can decrease the severity of *E. tenella* infection in broiler chickens.

Zinc influences the hormonal regulation of cell division. Specifically, the pituitary growth hormone (GH)-insulin-like growth factor-I axis is responsive to zinc status. Decreased GH concentrations have been observed in zinc deficiency, suggesting that zinc deficiency results in growth inhibition in animals [22]. Zinc is a component of multiple enzyme systems that control DNA synthesis, normal growth, reproduction, and wound healing [10]. In poultry, zinc minimized intestinal damage by *Eimeria* and reduced oocyst output [19]. Dietary zinc may also influence the immune system indirectly by interacting with the growth and infectivity of organisms and improving thymocyte levels, peripheral T-cell counts, and interferon levels of *Eimeria* spp. infected broilers [12–14]. Organic zinc had a significant impact in decreasing the intestinal inflammatory response by down-regulating the expression of pro-inflammatory cytokines, and organic zinc has been shown to reduce intestinal permeability caused by a *Clostridium perfringens* challenge in addition to coccidiosis [23, 24]. Zinc reduced oxidative stress and participated in the antioxidant defense system, which reduced the severity of *Eimeria* infection by ameliorating the degree of intestinal lipid peroxidation [12, 25].

There have been prior studies of zinc supplementation combined with drugs in humans. Zinc supplementation combined with antidepressant drugs has been shown to reduce the symptoms of depression, and zinc combined with antibacterial agents showed a synergistic inhibiting effect on intraoral volatile sulfur compounds [26, 27]. In poultry, there have been several studies using minerals for alternative coccidiosis control, but there have not been any previous studies on zinc or mineral and drug combinations for the control of coccidiosis or diseases in poultry. Many anticoccidial drugs are used in the poultry industry, and TOL is just one of them. The current approach to delay the onset of drug resistance is to employ rotational programs with different anticoccidial drugs combined with good husbandry, chemoprophylaxis, and vaccination [28]. Therefore, the effect of ZnOHCl supplementation combined with other drugs should be tested.

## Conclusion

This study demonstrated that ZnOHCl could suppress *E. tenella* oocyst sporulation. Zinc hydroxychloride supplementation was effective only for oocyst output reduction. Growth performance, lesion scores, and oocyst output were affected by ZnOHCl + TOL supplementation. These results indicate that ZnOHCl supplementation combined with the anticoccidial drug TOL improved growth performance and decreased the severity of *E. tenella* infection. Further study should be performed to investigate the efficacy of ZnOHCl supplementation combined with other anticoccidial drugs.

## Authors’ Contributions

TN and TC: Designed the study, material preparation, data collection, and analysis. TN: Drafted the manuscript. Both authors have read, reviewed, and approved the final manuscript.
